# Preventive and therapeutic effects of thymosin β4 N-terminal fragment Ac-SDKP in the bleomycin model of pulmonary fibrosis

**DOI:** 10.18632/oncotarget.8409

**Published:** 2016-03-27

**Authors:** Enrico Conte, Evelina Fagone, Elisa Gili, Mary Fruciano, Maria Iemmolo, Maria Provvidenza Pistorio, Daniela Impellizzeri, Marika Cordaro, Salvatore Cuzzocrea, Carlo Vancheri

**Affiliations:** ^1^ Department of Clinical and Experimental Medicine, University of Catania, 95124 Catania, Italy; ^2^ Department of Clinical and Experimental Medicine and Pharmacology, School of Medicine, University of Messina, 98166 Messina, Italy

**Keywords:** Ac-SDKP, lung fibrosis, bleomycin, mouse, IPF

## Abstract

In this study, the bleomycin model of pulmonary fibrosis was utilized to investigate putative anti-fibrotic activity of Ac-SDKP *in vivo*. Male CD-1 mice received intra-tracheal bleomycin (BLEO, 1 mg/kg) instillation in the absence or presence of Ac-SDKP (a dose of 0.6 mg/kg delivered intra-peritoneally on the day of BLEO treatment, d0, followed by bi-weekly additional doses). To evaluate therapeutic effects in a subset of mice, Ac-SDKP was administered one week after BLEO instillation (d7). Animals were sacrificed at one, two, or three weeks later. Measurement of fluid and collagen content in the lung, Broncho Alveolar Lavage Fluid (BALF) analysis, lung histology, immunohistochemistry (IHC), and molecular analysis were performed. Compared to BLEO-treated mice, animals that received also Ac-SDKP (at both d0 and d7) had significantly decreased mortality, weight loss, inflammation (edema, and leukocyte lung infiltration), lung damage (histological evidence of lung injury), and fibrosis (collagen histological staining and soluble collagen content in the lung) at up to 21 days. Moreover, IHC and quantitative RT-PCR results demonstrated a significant decrease in BLEO-induced IL-17 and TGF-β expression in lung tissue. Importantly, α-SMA expression, the hallmark of myofibroblast differentiation, was also decreased. This is the first report showing not only a preventive protective role of Ac-SDKP but also its significant therapeutic effects in the bleomycin model of pulmonary fibrosis, thus supporting further preclinical and clinical studies.

## INTRODUCTION

Idiopathic pulmonary fibrosis (IPF) is a progressive fibroproliferative disorder with poor prognosis similar to lung cancer. Very few pharmacological options are available, and the median survival time is only 3–5 years following diagnosis. The etiology of this fibrotic lung disease is by definition unknown but an understanding of IPF pathophysiology over the last two decades has shifted from a chronic inflammatory process to an abnormal wound healing cascade with aberrant fibroblast/myofibroblast proliferation and accumulation of extracellular matrix (ECM) proteins such as collagen [1, 2].

N-acetyl-seryl-aspartyl-lysyl-proline (Ac-SDKP) is an endogenous (normally present in organs and in biological fluids) tetrapeptide generated by prolyl oligopeptidase (POP) from its precursor thymosin-β4 (Tβ4) [3]. Ac-SDKP is, in turn, a natural substrate of the N-domain of angiotensin-converting enzyme (ACE), cleaving this peptide into inactive fragments [4].

Several studies have demonstrated that Ac-SDKP may have antifibrotic properties. Administration of Ac-SDKP in rats prevents, and even reverses, collagen deposition in a model of myocardial infarction [5]. Consistent antifibrotic effects of Ac-SDKP have been shown in experimental liver [6–7] and kidney fibrosis [8–9]; Ac-SDKP also inhibits pulmonary fibrosis in rats with SiO2-induced silicosis [10, 11].

BLEO is a potent anti-tumor agent for several neoplasms, but its administration is limited by drug-induced lung fibrosis. Therefore, BLEO is one of the most widely used drugs for inducing lung fibrosis in animals, due to its ability to provoke a histological lung pathology similar to that described in patients undergoing chemotherapy, and in part overlapping with IPF feature. BLEO damage to the lungs is characterized by patchy parenchymal inflammation, basement membrane and alveolar epithelial cell injury with reactive hyperplasia, epithelial-mesenchymal transition, activation, and differentiation of fibroblasts to myofibroblasts. Although it is known that the BLEO animal model of IPF fails to reflect some of the histological and pathological features typical of the human disease, it is widely accepted that this model sufficiently recapitulates the hallmark characteristics of the IPF [12]. Thus in this study, we utilized CD-1 mice treated with an intra-tracheal instillation of bleomycin to study the putative preventive and therapeutic anti-fibrotic effects of Ac-SDKP in the lung. Also, in order to investigate mechanisms, we assessed the effects of Ac-SDKP on the expression of IL-17, a cytokine pivotal in BLEO-induced lung damage and fibrosis, and α-SMA, the hallmark of myofibroblast differentiation.

## RESULTS

### Ac-SDKP co-treatment reduced BLEO-induced mortality and weight loss

**Figure 1:**
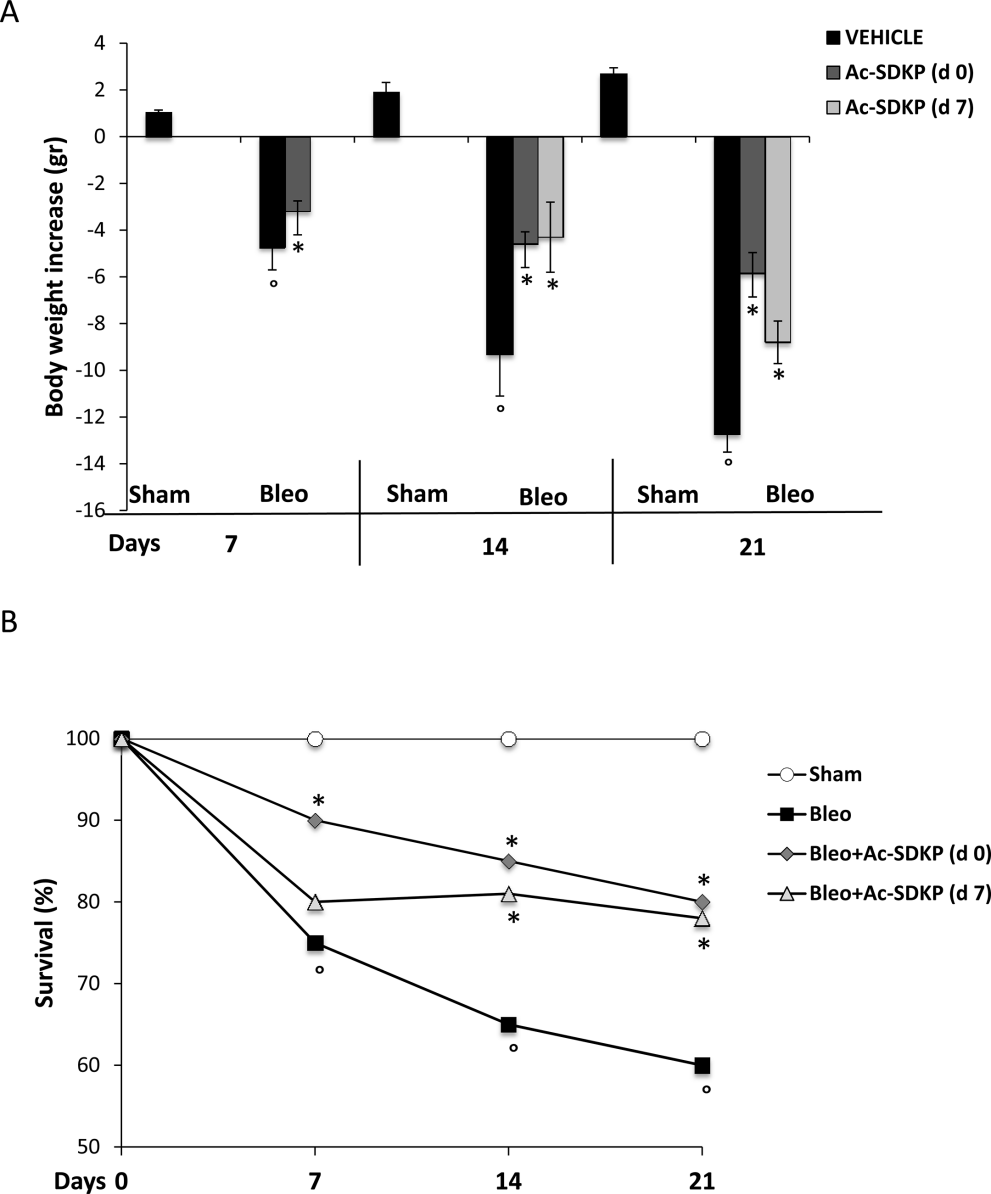
Protective effects of Ac-SDKP treatment on BLEO-induced mortality and weight loss (**A**) Body weight loss/gain expressed in grams. The zero line was considered the mean value of the original mouse weight. Groups/treatments/time points are indicated. The asterisk indicates a *P* < 0.05 versus Bleo, the circle a *P* < 0.05 versus Sham. (**B**) Mortality rate expressed as % of survival. Groups/treatments/time points are indicated.°*P* < 0.05 versus Sham, *P < 0.05 versus Bleo.

As shown in Figure 1A and 1B, the BLEO-induced mortality and body weight loss was significantly reduced in mice that had received Ac-SDKP both immediately (d0) and late(d7) after BLEO instillation. A significantly increased survival of Ac-SDKP co-treated mice was observed at 7 days (Ac-SDKP 90% vs BLEO 75% *P* < 0.05), 14 days (Ac-SDKP d0 85%, d7 81% vs BLEO 65% *P* < 0.05 both) and 21 days (Ac-SDKP d0 80%, d7 78% vs BLEO 60% *P* < 0.05 both), Figure 1A. Similarly, as show in Figure 1B, the substantial decrease in body weight induced by BLEO instillation was significantly prevented at 7 days (Ac-SDKP −3.2 ± 0.45 vs BLEO −4.75 ± 0.95 *P* < 0.05), 14 days (Ac-SDKP d0 −4.6 ± 0.53, d7 −4.3 ± 1.5 vs BLEO −9.3 ± 1.8 *P* < 0.05 both), and 21 days (Ac-SDKP d0 −5.86 ± 0.9, d7 −8.8 ± 0.91vs BLEO −12.7 ± 0.8 *P* < 0.05 both) in mice co-treated with Ac-SDKP at both d0 and d7.

### BLEO-induced leukocyte infiltration and lung edema were significantly reduced by Ac-SDKP co-treatment

**Figure 2:**
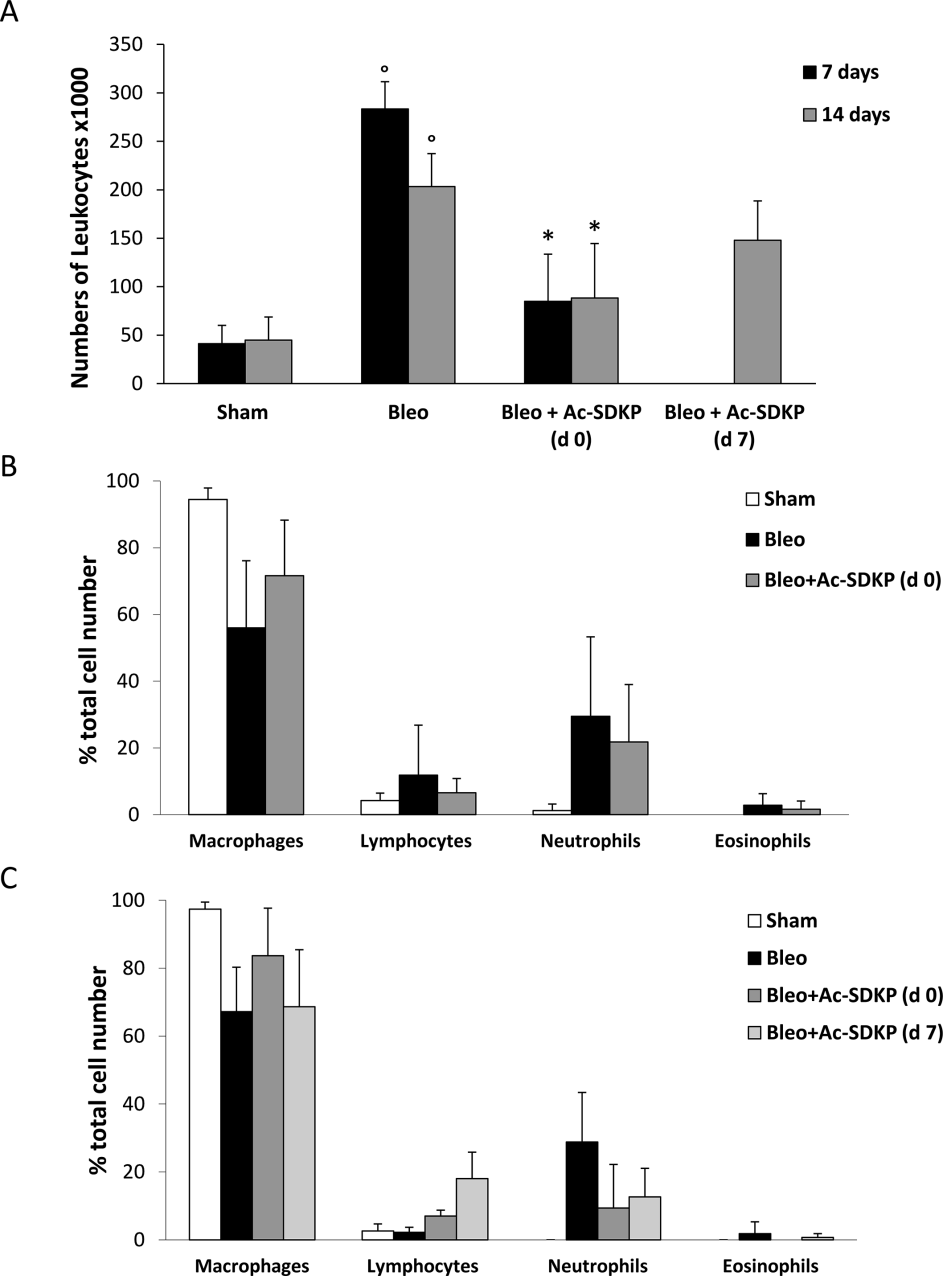
Protective effects of Ac-SDKP treatment on BLEO-induced leukocyte lung infiltration (**A**) Cellularity in BALF of indicated mice groups, evaluated by a hemocytometer where cells were visualized with trypan blue staining. The total number of leukocytes, ×10^3^ per milliliter (mean ± s.d), is reported for each group (at least five mice analyzed) at the indicated time points.°*P* < 0.05 versus Sham, *P < 0.05 versus Bleo. (**B**) Differential cell counts in cytospins prepared from BALF at 7 days were obtained by observing a total of 100 cells in randomly chosen fields of each sample. (**C**) Differential cell counts in cytospins prepared from BALF at 14 days.

The BLEO-induced inflammatory response evaluated by the presence of leukocytes in BALF as well as by lung edema at 7 and 14 days was significantly inhibited in mice treated with Ac-SDKP. As shown in Figure 2A, the large increase in the number of leukocytes found in BALF of BLEO-treated mice was significantly reduced by Ac-SDKP co-treatment at d0, and substantially arrested by delayed Ac-SDKP treatment (BALF total cell number, ×10^3^ per ml at 7 days: Ac-SDKP 85 ± 49 vs BLEO 283,3 ± 28 *P* < 0.05; and 14 days: Ac-SDKP d0 88.3 ± 56, d7 148 ± 41 vs BLEO 203,3 ± 34 *P* < 0.05 and *P* > 0.05, respectively). Moreover, Ac-SDKP co-treatment substantially halted the BLEO-induced increase in the number of neutrophils at both 7 and 14 days, as shown in Figure 2B and 2C, respectively.

In addition, data in Table 1 demonstrate that the relevant BLEO-induced lung edema observed in mice sacrificed at both 7 and 14 days was significantly reduced by Ac-SDKP co-treatment at both d0 and d7.

**Table 1:** The wet/dry lung weight mean (± s.d.) ratio of at least three mice for each group is reported

Wet / dry lung weight ratio				
	Sham	Bleo	Bleo + Ac-SDKP (d 0)	Bleo + Ac-SDKP (d 7)
**7 days**	3,3 (± 0,1)	8,15 (± 0,85)°	5,05 (± 0,15)***	
**14 days**	3,37 (± 0,22)	10,2 (± 0,4)°	6,05 (± 0,35)**	7,35 (± 0,15)*

* *P* < 0.05 versus Sham

** *P* < 0.01 versus Sham

*** *P* < 0,001 versus Sham;°*P* < 0,05 versus Bleo.

### Marked histological signs of BLEO-induced lung damage and fibrosis were significantly reduced by Ac-SDKP co-treatment

**Figure 3:**
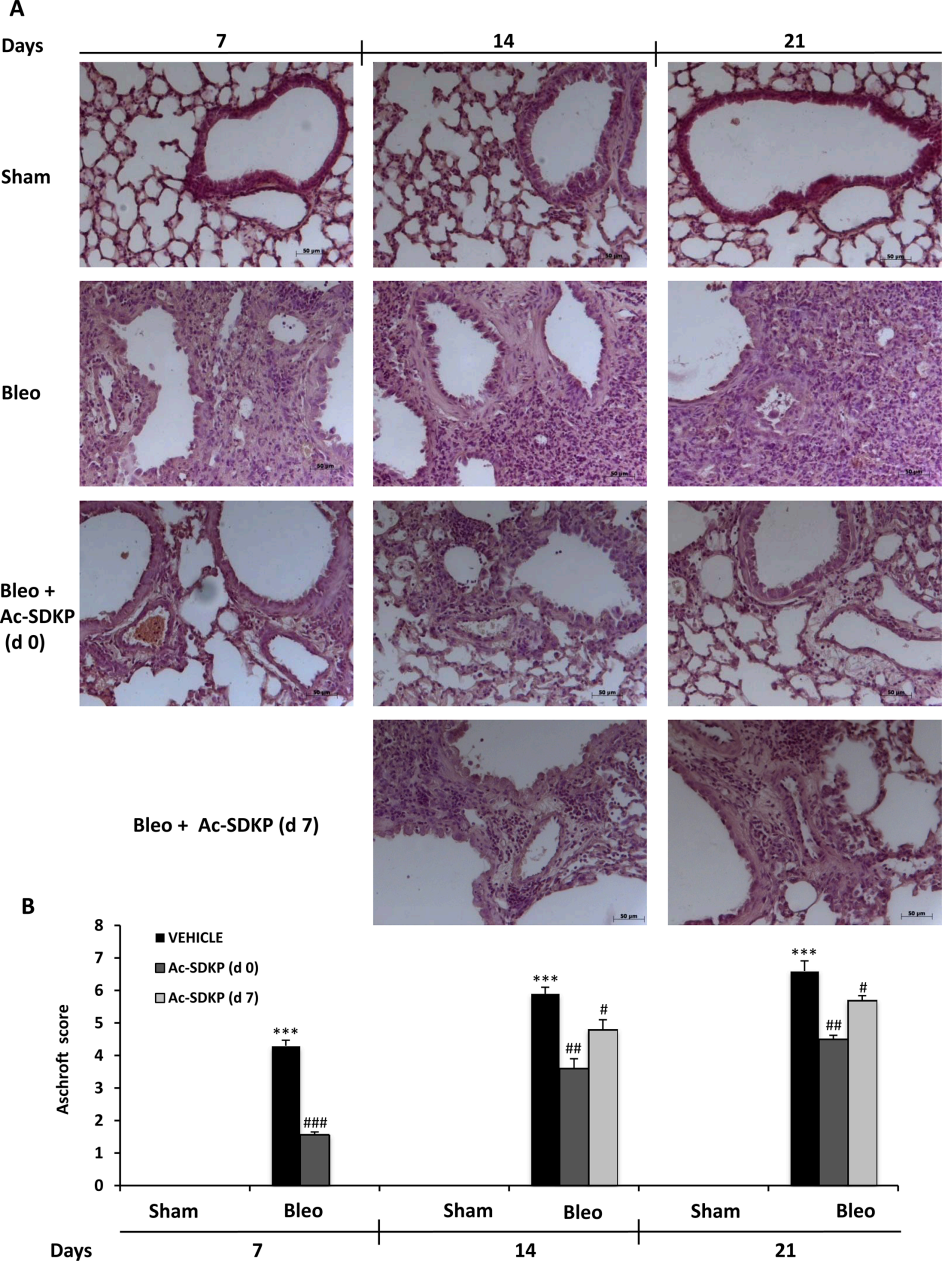
Ac-SDKP treatment suppressed BLEO-induced histological marks of lung damage and fibrosis in mouse lung (**A**) Representative microphotographs (150 ×)of FFPE lung tissue slices stained by H & E from indicated mice at the indicated time points. (**B**) Semi-quantitative fibrosis scoring was assessed according to the Ashcroft scale. Slides were scored by a single investigator in a blinded fashion. Graphs show mean values ± s.d. of at least five mice for each group. *P < 0,05 vs Sham; **P < 0,01 vs Sham; ***P < 0,001 vs Sham; #*P* < 0,05 vs Bleo; ##*P* < 0,01 vs Bleomycin; ###*P* < 0,001 vs Bleo.

**Figure 4:**
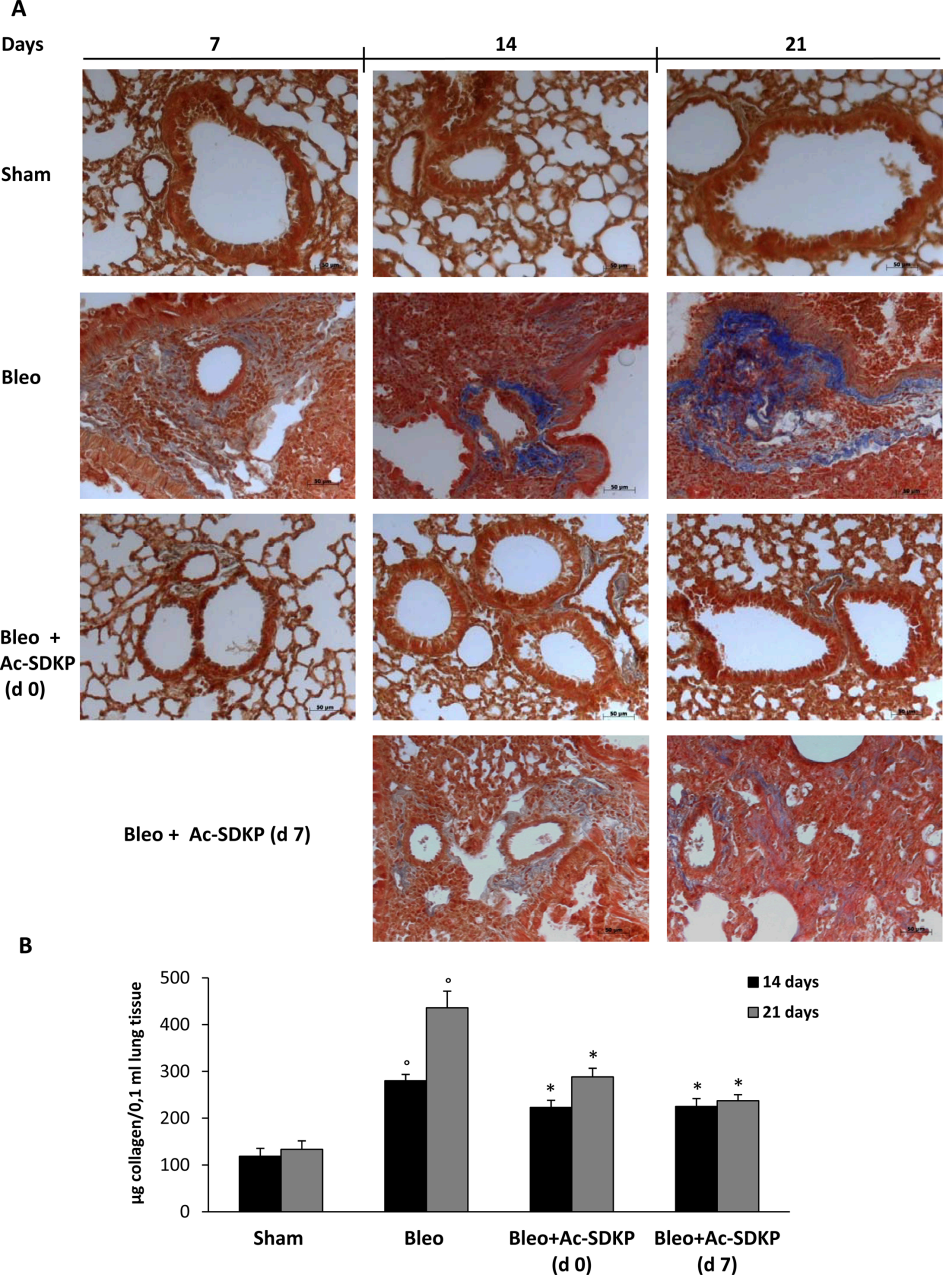
Effects of Ac-SDKP treatment on BLEO-induced increase in collagen deposition in mouse lung tissue (**A**) Representative microphotographs (150x) of FFPE lung tissue slices stained with Masson's trichrome that is specific for collagen detection (blue). (**B**) Lung content (μg) of soluble collagen measured by the Sircol Soluble Collagen Assay in homogenized tissues (0.1 ml) of at least five mice for each group. Graphs report mean values ± s.d.,°*P* < 0.05 versus Sham, *P < 0.05 versus Sham group.

As shown in left panels of Figure 3A, following 7 days of BLEO instillation, histological examination of the lung tissue revealed an extensive inflammatory infiltration by leukocytes extending through the lung epithelium with granulomas in the perivascular region as well as moderate fibrous thickening of the alveolar/bronchiolar walls. Moreover, the initial extracellular collagen deposition was evidenced by Masson's staining (Figure 4A). In contrast, preventive Ac-SDKP co-treatment suppressed both the inflammatory response and the ongoing fibrotic process, as recapitulated by the Ashcroft score in Figure 3B: Ac-SDKP 1.56 ± 0.09 vs BLEO 4.3 ± 0.17 *P* < 0.001). Established fibrosis and severe distortion of the lung structure in the BLEO-treated mice at 14 and 21 days, as shown in middle and right panels of Figures 3A and 4A, were significantly reduced not only in mice cotreated with Ac-SDKP since dO but also in the animals administered Ac-SDKP after 7 days following BLEO instillation (Ashcroft score; at 14 days: Ac-DSKP d0 3.6 ± 0.3, d7 4.8 ± 0.3 vs BLEO 5.9 ± 0.2 *P* < 0.01 and *P* < 0.05 respectively; and 21 days: Ac-SDKP d0 4.5 ± 0.12, d7 5.7 ± 0.14 vs BLEO 6.6 ± 0.31 *P* < 0.01 and *P* < 0.05 respectively). Since there was no progression of the fibrotic process in animals with delayed Ac-SDKP co-treatment, these findings demonstrate a therapeutic effect of Ac-SDKP.

### Ac-SDKP co-treatment reduced the BLEO-induced increase of collagen content in the lung

As shown in Figure 4A, representative microphotographs of FFPE lung tissue slices stained with Masson's trichrome specific for collagen fibers indicate the substantial prevention of BLEO-induced extracellular collagen deposition in mice co-treated with Ac-SDKP. These results are paralleled by quantitative measurement of total soluble collagen in the lung tissue of mice sacrificed at 14, or 21 days. As shown in Figure 4B, compared to BLEO-treated control mice, a significant reduction in the collagen increase was observed in Ac-SDKP cotreated mice, at both time points (Collagen μg/0.1 ml at 14 days: Ac-SDKP d0 223.04 ± 15.21, d7 225.04 ± 16.99 vs BLEO 280.04 ± 13.58 *P* < 0.05 both; and at 21 days:Ac-SDKP d0 288.36 ± 18.39, d7 237.28 ± 13.02 vs BLEO 436,12 ± 35.62 *P* < 0.05 both).

### BLEO-induced over-expression of IL-17 and TGF-β in the lung tissue was inhibited in BLEO/Ac-SDKP co-treated mice

**Figure 5:**
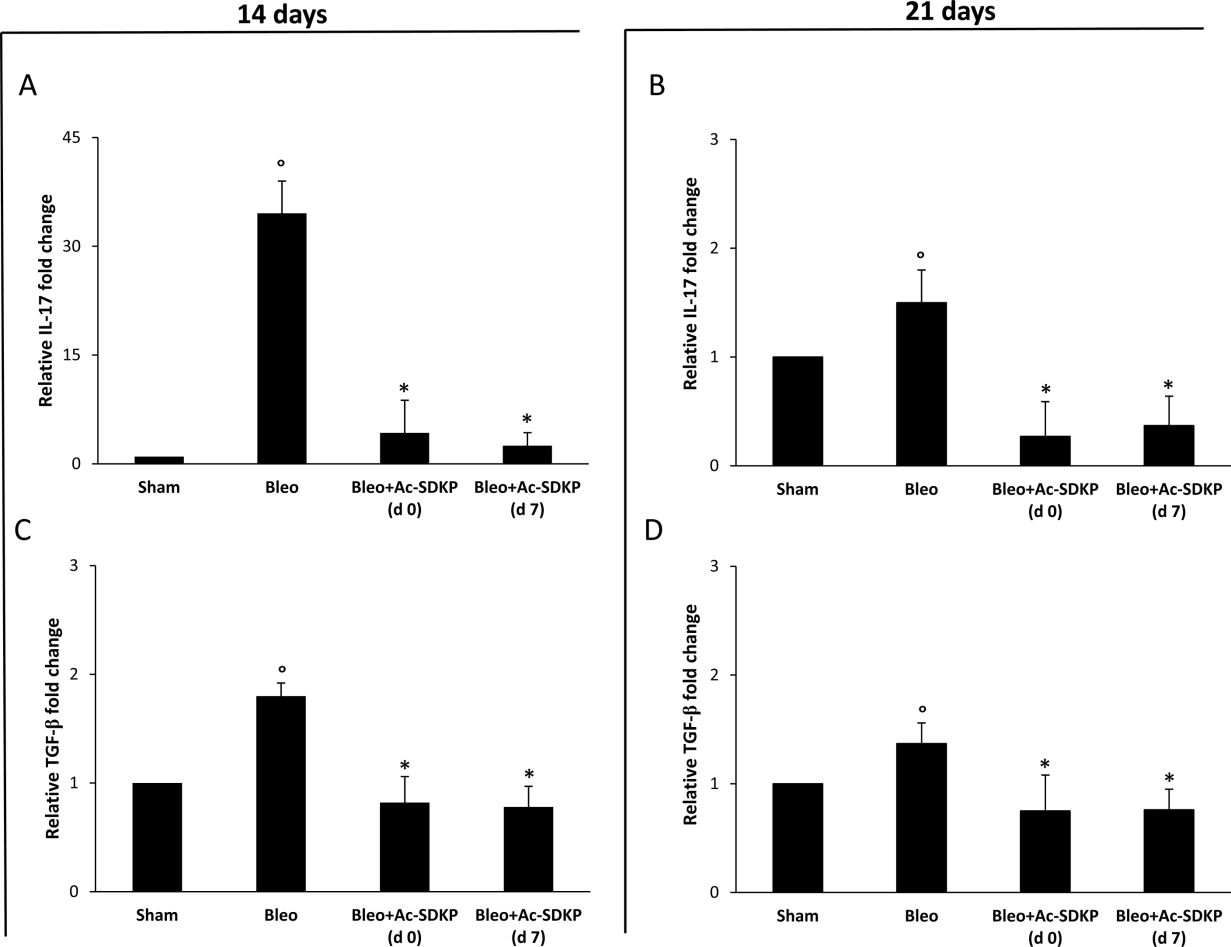
Ac-SDKP treatment suppressed BLEO-induced over-expression of IL-17 and TGF-β mRNA in mouse lung tissue Q-RT-PCR analysis of IL-17 (**A** and **B**) and TGF-β (**C** and **D**) mRNA expression in lung tissue. Reported values are means ± s.d. of the relative fold inductions calculated with the ΔΔCt method considering Sham values as calibrator, i.e. expression level = 1). Three replicates of at least three separate RNA samples from different mice (*N* = 5) for each group were performed. *P < 0.05 versus Sham, °*P* < 0.05 versus Sham group.

As shown in Figure 5A and 5C, molecular analysis of IL-17 and TGF-β expression demonstrates that at 14 days in the lung homogenates of Ac-SDKP co-treated mice (both at d0 and d7) the near 35-fold increase in IL-17 mRNA expression as well as the near 1, 8-fold increase in TGF-β mRNA expression observed in the lungs of BLEO-treated mice were completely prevented. Also at 21 days the BLEO-induced over-expression of both cytokines was significantly inhibited in Ac-SDKP co-treated mice (Figure 5B and 5D).

**Figure 6:**
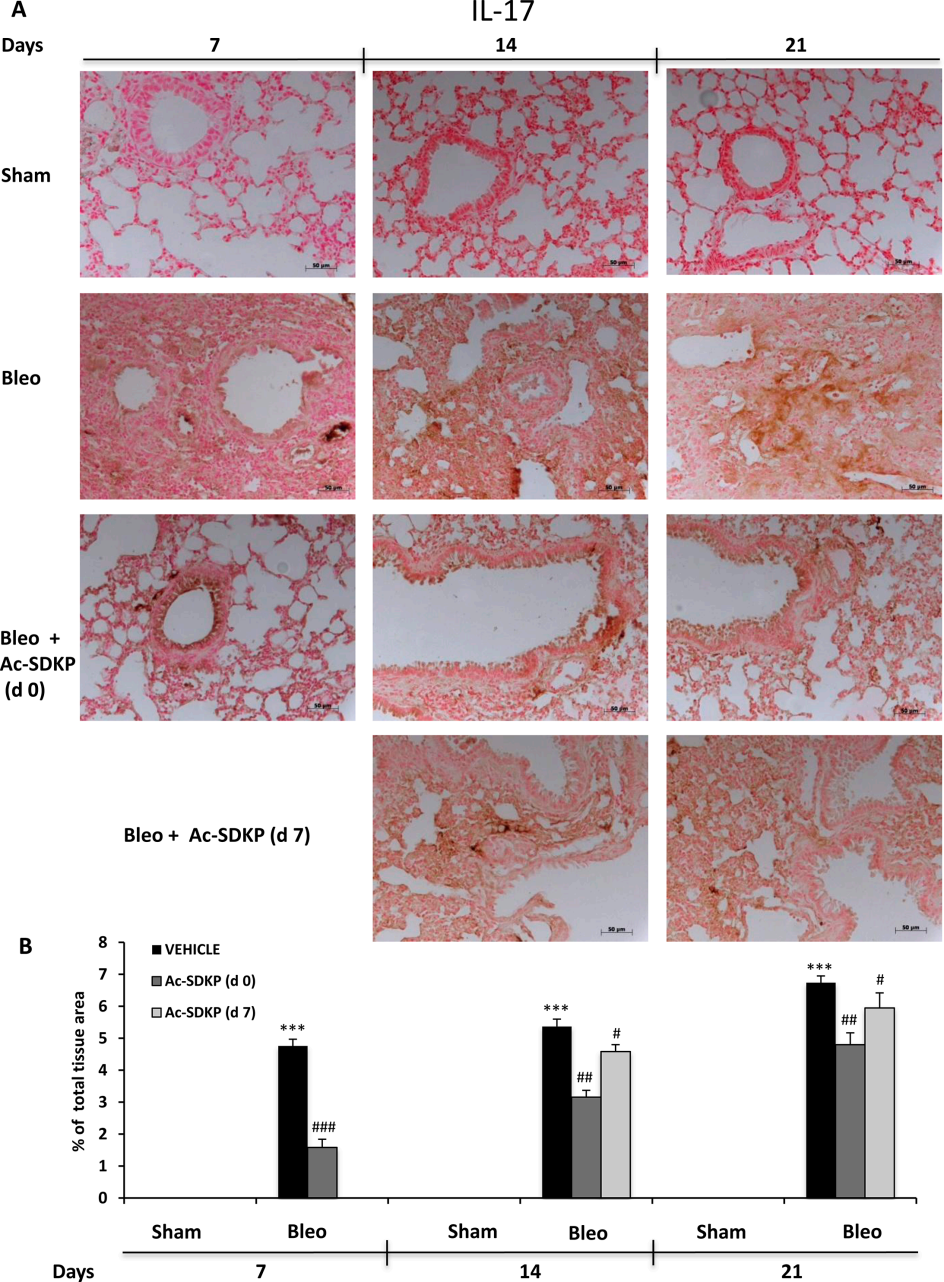
Ac-SDKP treatment inhibited BLEO-induced IL-17 expression in mouse lung tissue (**A**) Representative microphotographs of IL-17 IHC in FFPE lung tissue slices from the indicated mice/groups/time points using an anti-mouse IL-17 rabbit polyclonal antibody. (**B**) Densitometric analysis of the microphotographs using Optilab Graftek software: positive areas are expressed as percentage of total tissue area. The asterisks indicate a *P* < 0.05 versus Sham, ###*P* < 0.05 versus Bleomycin

**Figure 7:**
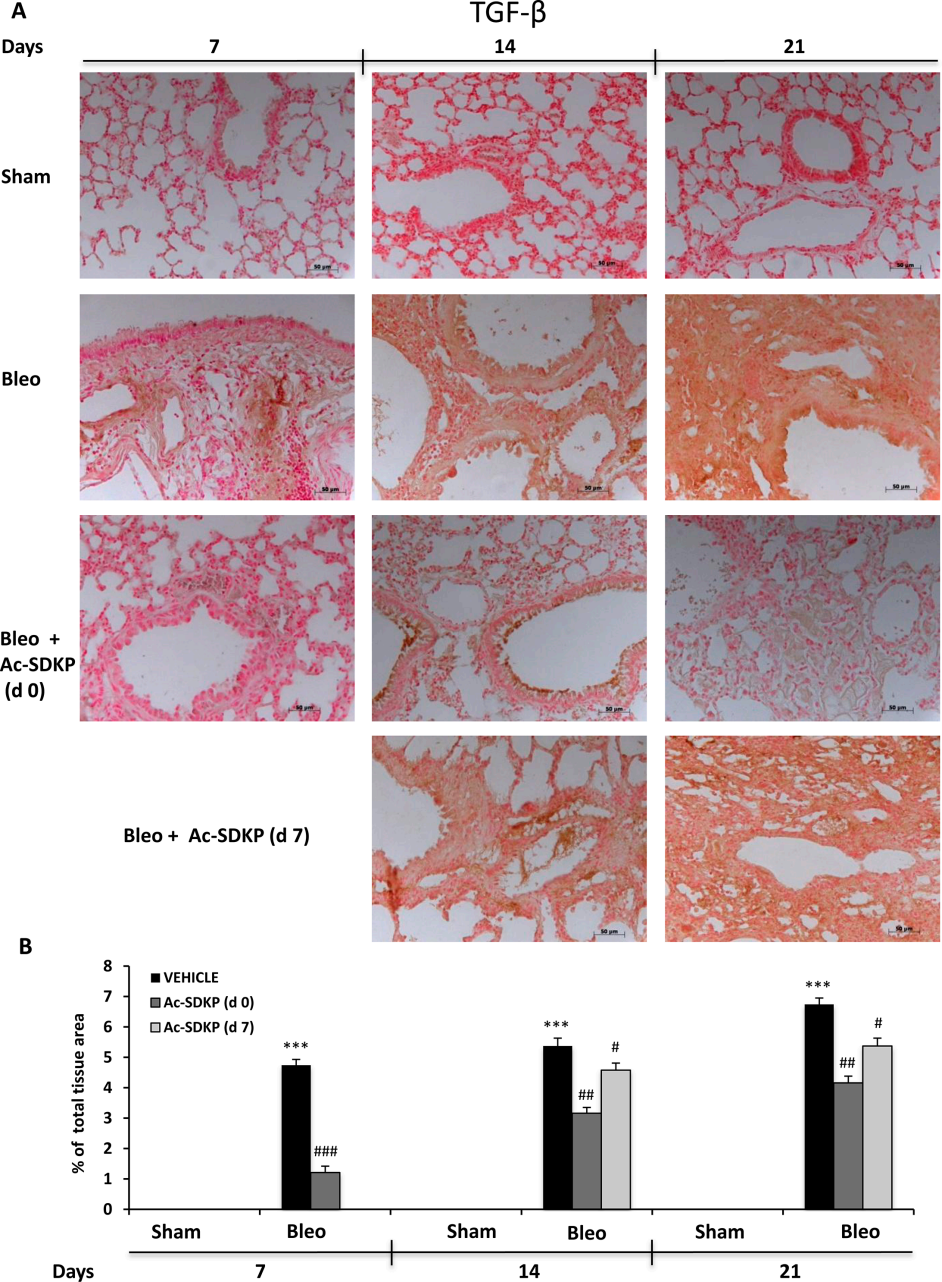
Ac-SDKP treatment inhibited BLEO-induced TGF-β expression in mouse lung tissue (**A**) Representative microphotographs of TGF-β IHC in FFPE lung tissue slices from the indicated mice/groups/time points by using an anti-mouse TGF-β rabbit polyclonal antibody. (**B**) Densitometric analysis of the microphotographs using Optilab Graftek software: positive areas are expressed as percentage of total tissue area. The asterisks indicate a *P* < 0.05 versus Sham, ###*P* < 0.05 versus Bleomycin.

In addition, IL-17 and TGF-β IHC analysis in the lung tissue slices shown in Figures 6A and 7A, respectively, demonstrate no positivity for both cytokines in Sham mice whereas a strong signal was detected in samples of BLEO-treated mice where stromal, interstitial and infiltrating inflammatory cells, and also epithelial cells appear intensively positive for both cytokines. In contrast, these intense immunoreactivities were significantly reduced in Ac-SDKP co-treated mice (both at d0 and d7), Figures 6B and 7B.

### Ac-SDKP co-treatment was able to inhibit BLEO-induced over-expression of α-SMA in the lung tissue

**Figure 8:**
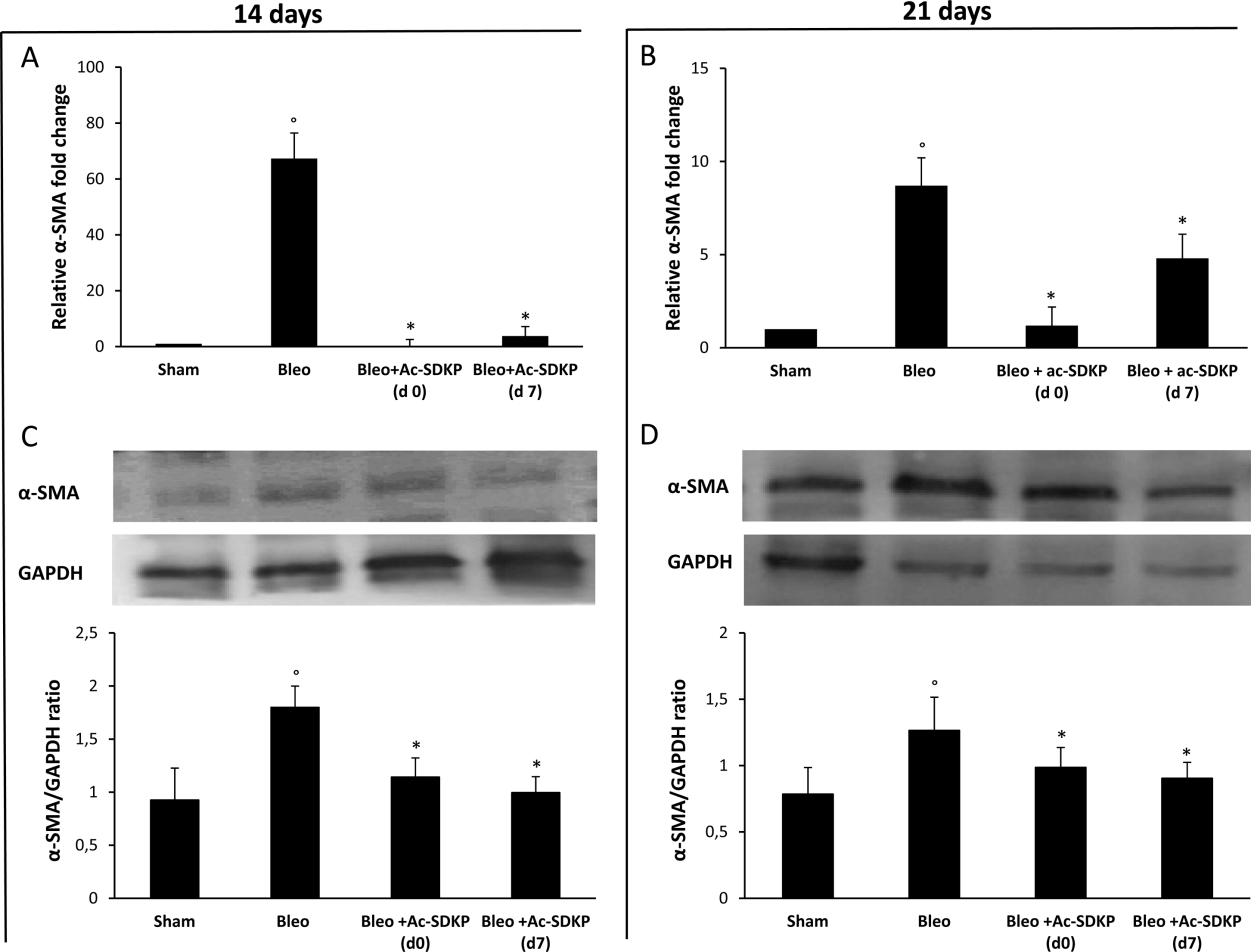
Ac-SDKP treatment inhibited the BLEO-induced α-SMA expression in mouse lung tissue at mRNA and protein level Q-RT-PCR analysis of α-SMA mRNA expression in lung tissue. Reported values are means ± s.d. of the relative fold inductions calculated with the ΔΔCt method considering Sham values as the calibrator, i.e. expression level = 1). Three replicates of at least three separate RNA samples from different mice for each group (*N* = 5) were performed. (**B**) Representative Western blot analysis of α-SMA protein expression and quantitation with mean values ± s.d. of α-SMA/GAPDH ratios of three separate experiments with tissue lysates from different mice (*N* = 5) for each group. *P <0.05 versus Sham, °*P* < 0.05 versus Sham group.

As shown in Figure 8A, the strong BLEO-induced α-SMA mRNA over-expression (more than 60× fold change) in the lung tissue at day 14 was completely blocked in co-treated mice which received Ac-SDKP at both d0 and d7. In agreement with this finding, Western blot analysis shown in Figure 8C demonstrates a significant reduction of α-SMA protein in the lung tissue of BLEO/AC-SDKP treated mice. At days 21, as shown in Figure 8B and 8D, α-SMA mRNA over-expression was much reduced in BLEO-treated mice (less than 10× fold change), probably due to being completion of myofibroblast differentiation, nevertheless both α-SMA mRNA and protein expression were significantly further reduced in the AC-SDKP co-treated mice (both at d0 and d7).

## DISCUSSION

Previously we showed anti-fibrotic properties of Ac-SDKP *in vitro*, differently to its precursor Tβ4 [16]. On the other hand, we demonstrated that Tβ4 was unable to prevent established pulmonary fibrosis at 14 and 21 days in BLEO-treated mice, in contrast to its protective role at 7 days [17]. In the current study we utilized the identical mouse model of the previous research (same strain, BLEO dose and instillation mode) to investigate *in vivo* the effects of exogenous Ac-SDKP (same drug molar concentration, route and scheduling of Tβ4) by comparison with Tβ4, Here we demonstrated that, besides protective effects on mortality, body weight loss and inflammation, Ac-SDKP carried out both preventive and therapeutic actions against lung damage and fibrosis at up to 21 days, as determined by histological score and collagen content in the lung. These findings are in line with our previous observations *in vitro* and reinforce the anti-fibrotic function of Tβ4 amino-terminal active site containing the four amino acids Ac-SDKP, which can be released from the parent molecule (both endogenous and exogenous) via processing and/or degradation of intact Tβ4 [18]. Accordingly, Zuo and colleagues showed that Ac-SDKP has anti-fibrotic effects in both early and late stages of renal fibrosis induced by unilateral ureteral obstruction whereas Tβ4 alone has context- and time-dependent effects on renal fibrosis [9]. Furthermore, the current results are in agreement with observations of a previously published study showing that accumulation of endogenous Ac-SDKP in ACE N-terminal catalytic site-KO mice alleviates BLEO-induced lung injury, in particular fibrosis [19]. Similarly, exogenous Ac-SDKP has been shown to inhibit pulmonary fibrosis in rats with SiO2-induced silicosis [10, 11]. Previous experiments with knock-out mice demonstrated that IL-17 is essential for BLEO-induced fibrosis [20], and it was shown that recombinant IL-17 is able to induce fibrosis [20]. Furthermore, blocking IL-17 promoted the resolution of pulmonary inflammation and fibrosis [21]. Based on these findings, we also investigated the effects of Ac-SDKP on IL-17 expression. Q-RT-PCR and IHC data demonstrated a significant inhibition of BLEO-induced IL-17 expression in lung tissue of Ac-SDKP co-treated mice, including when AC-SDKP was therapeutically administered one week after BLEO instillation. Moreover, since myofibroblast differentiation, characterized by α-smooth muscle actin (α-SMA) expression, is thought to be the key process in organ fibrosis [22], and it is induced by TGF-β released by damaged epithelial cells and/or by infiltrating inflammatory cells [23], in this study we examined whether Ac-SDKP can inhibit TGF-β signaling and myofibroblast differentiation. Based on q-RT-PCR and IHC results, we show that both initial and delayed Ac-SDKP treatment was able to significantly decrease the BLEO-induced TGF-β over-expression in the damaged lung. Importantly, we also found that both preventive and therapeutic treatment with Ac-SDKP reduced fibroblast/myofibroblast differentiation, as demonstrated by qRT-PCR and Western blot analysis of α-SMA expression in the lung tissue. Accordingly, it was previously demonstrated that Ac-SDKP can inhibit the myofibroblast differentiation of human fetal lung fibroblasts (MRC-5) cells induced by Ang II [24]. Furthermore, it was shown that Ac-SDKP suppresses epithelial-mesenchymal transition in A549 cells via HSP27 signaling [25]. Since effective IPF therapies should be focused on halting the fibroblast/myofibroblast differentiation process, and consequently abnormal tissue remodeling as well as excessive accumulation of extracellular matrix, we propose Ac-SDKP as a valid putative drug for lung fibrosis. Moreover, it is noteworthy that in this comparative study Ac-SDKP was administered, as previously Tβ4, bi-weekly and at a middle dosage yet it was able to block the established lung damage and the ongoing fibrosis also administered one week after BLEO instillation. One can only speculate whether a real therapeutic scheduling (e.g. daily vs bi-weekly) at a higher dosage could not only block but also reverse the pathologic process. However, considering the potential role of Ac-SDKP in some malignancies [26–29], even though controversial [30], caution should be used to determine whether Ac-SDKP can be harmful in a subset of patients. Nevertheless we must seriously consider the potential therapeutic utilization of Ac-SDKP against pulmonary fibrosis. Further preclinical and clinical studies are warranted.

## MATERIALS AND METHODS

### Materials

Ac-SDKP was purchased from Bachem, Peninsula Laboratories, San Carlos, CA, USA. Unless otherwise stated, all other chemicals were obtained from Sigma-Aldrich Company Ltd. (Poole, Dorset, U.K.) at the highest commercial grade available. All stock solutions were prepared in non-pyrogenic saline (0.9% NaCl; Baxter, Italy, UK).

### Animals and *in vivo* experiments

Male CD-1 mice (Harlan Nossan; Italy) were housed in a controlled environment and provided with standard rodent chow and water. Animals were randomly allocated into the following groups: (i) Bleo (*N* = 24). Mice were subjected to BLEO instillation and received saline intra-peritoneally (i.p.), immediately after BLEO and bi-weekly until days 7, 14, or 21, (ii) Bleo + Ac-SDKP (*N* = 24). Same as the Bleo group but Ac-SDKP was administered (0.6 mg/kg) i.p., immediately after BLEO and bi-weekly until day 7, 14, or 21, (iii) Bleo + Ac-SDKP (delayed) group (*N* = 16). Sham-operated group in which identical procedures to the Bleo group were performed, except that saline was used instead of bleomycin and Ac-SDKP was administered one week after BLEO and bi-weekly until day 14 or 21. (iv) Sham group (*N* = 15). Sham-operated group in which an identical procedure to the Bleo group was performed, except that saline was used instead of BLEO and the vehicle for Ac-SDKP was given immediately after BLEO and bi-weekly until sacrifice. Mice were euthanized with a pentobarbitone overdose.

### BALF analysis

After sacrifice mouse trachea were immediately cannulated with an intravenous polyethylene catheter equipped with a 24-gauge needle on a 1-ml syringe. Lungs were washed once with 0.5 ml D-PBS (GIBCO, Paisley, UK). The BALF was spun at 800 rpm, the supernate was removed, and the pelleted cells were resuspended in PBS. BALF cells were stained with trypan blue and counted with a hemocytometer Cytospins were prepared from resuspended BALF cells by centrifuging 50,000 cells onto microscope slides using a Shandon Cytospin 3 (Shandon, Astmoore, UK). Slides were allowed to air dry and were then stained with Diff-Quick Stain Set (Diff-Quick; Baxter Scientific, Miami, FL). Using optical microscopy, a total of 100 cells were examined from each sample in randomly chosen fields.

### Measurement of water content in the lung

The wet weight of one lung was measured after careful excision of extraneous tissues. The lung was then heated at 180°C for 48 hr and afterwards the dry weight was measured as previously described [13], and wet/dry weight ratio was calculated.

### Soluble collagen assay

The total content of soluble collagen in the lung tissue was measured using the Sircol Soluble Collagen Assay (Biocolor, Newtownabbey, Northern Ireland) as previously described [14].

### Histology

Lung biopsies were fixed in 10% (w/v) PBS-buffered formaldehyde solution at room temperature, dehydrated using graded ethanol, and embedded in Paraplast (Sherwood Medical, Mahwah, NJ, USA). Sections (8-μm) were then deparaffinized with xylene, stained with either conventional hematoxylin & eosin (H & E) or by the Masson's method for collagen detection, and observed using a light microscope (Dialux 22 Leitz). The severity of lung damage and fibrosis was assessed according to the method of Ashcroft et al. [15] as previously described [14].

### Immunohistochemistry (IHC)

Formalin fixed paraffin-embedded (FFPE) lung sections (8-μm) were used for immunohistochemical localization of IL-17 and TGF-β. Endogenous peroxidase in de-paraffinized sections was quenched with 0.3% (v/v) hydrogen peroxide in 60% (v/v) methanol for 30 min. The sections were made permeable by incubation with 0.1% (w/v) Triton X-100 in PBS for 20 min. Non-specific adsorption was minimized by incubating the sections in 2% (v/v) normal goat serum in PBS for 20 min. Endogenous biotin and avidin binding sites were blocked by sequential incubation for 15 min with biotin and avidin, respectively. Sections were then incubated overnight at 4°C with anti-mouse TGF-β rabbit polyclonal antibody (Santa Cruz Biotechnology, 1:100 in PBS, v/v) or with anti-mouse IL-17 rabbit polyclonal antibody (Santa Cruz Biotechnology, 1:100 in PBS, v/v). Then, the sections were washed with PBS, and incubated with a biotin-conjugated goat anti-rabbit IgG secondary antibody followed by an avidin-biotin peroxidase complex (Vector Laboratories, Milan, Italy). The immunoreactions were visualized by incubating the sections four minutes in a 0.1% 3, 3′-diaminobenzidine (DAB) and 0.02% hydrogen peroxide solution (DAB substrate kit; Vector Laboratories). Densitometric analysis of microphotographs was performed by using Optilab Graftek software, and positive areas were expressed as percentage of the total tissue area.

### RNA extraction, reverse transcription and quantitative reverse transcriptasePCR (qRT-PCR)

Total RNA was extracted by using TRIZOL (Invitrogen, UK), quantified by UV spectroscopy (BIO-photometer, Eppendorf, Germany), and treated with DNAse (Invitrogen). cDNA was generated using Superscript II Reverse Transcriptase (Invitrogen) and random hexamer primers (Invitrogen) according to the manufacturer's instructions. qRT-PCR was performed using in a duplex assay the commercially available Taqman assays for mouse α-SMA (ACTA2), COL-I (COL-IA1), and the housekeeping GAPDH gene (QIAGEN, Germany, for the firsts, and Thermo Fisher, USA, for the last) following the manufacturer's instructions. Amplification conditions were identical for all genes: 94°C, 5 min and 45 cycles of 2 steps: 95°C, 15 sec and 60°C, 30 sec. Relative quantification of target gene levels was performed by comparing ΔCt as described elsewhere [15, 16], and calculating ΔΔCt with the values from the Sham as the calibrator.

### Western blot analysis

Cell lysates were subjected to denaturating SDS gel electrophoresis followed by electroblotting and incubation with either polyclonal rabbit anti-mouse α-SMA Ab (1:1000, Novus Biologicals, USA NB600-531), or monoclonal mouse anti-human GAPDH (1:1000, Merck Millipore) cross-reacting with the mouse protein.

### Statistical analysis

Where suitable, data were analyzed by one-way ANOVA followed by a Bonferroni post-hoc test for multiple comparisons. A *p*-value of less than 0.05 was considered significant. Analysis of survival data was performed by Fisher's exact probability test. The Mann–Whitney test was used to examine differences between the body weight and the organ weights of the control and experimental groups.
